# Immunomodulatory Effects of Pure Cylindrospermopsin in Rats Orally Exposed for 28 Days

**DOI:** 10.3390/toxins14020144

**Published:** 2022-02-15

**Authors:** Leticia Diez-Quijada, Antonio Casas-Rodriguez, Remedios Guzmán-Guillén, Verónica Molina-Hernández, Rafael G. Albaladejo, Ana María Cameán, Angeles Jos

**Affiliations:** 1 Area of Toxicology, Faculty of Pharmacy, Universidad de Sevilla, 41012 Seville, Spain; ldiezquijada@us.es (L.D.-Q.); acasasr@us.es (A.C.-R.); camean@us.es (A.M.C.); angelesjos@us.es (A.J.); 2 Department of Anatomy and Comparative Pathology and Toxicology, Faculty of Veterinary Medicine, Universidad de Córdoba, Campus de Rabanales, 14071 Córdoba, Spain; b62mohev@uco.es; 3 Department of Plant Biology and Ecology, Faculty of Pharmacy, Universidad de Sevilla, 41012 Sevilla, Spain; albaladejo@us.es

**Keywords:** cylindrospermopsin, immunotoxicity, mRNA expression, thymus, spleen, rats

## Abstract

Cylindrospermopsin (CYN) is a ubiquitous cyanotoxin showing increasing incidence worldwide. CYN has been classified as a cytotoxin and, among its toxic effects, its immunotoxicity is scarcely studied. This work investigates for the first time the influence of oral CYN exposure (18.75; 37.5 and 75 µg/kg b.w./day, for 28 days) on the mRNA expression of selected interleukin (IL) genes (IL-1β, IL-2, IL-6, Tumor Necrosis Factor alpha (TNF-α), Interferon gamma (IFN-γ)) in the thymus and the spleen of male and female rats, by quantitative real-time polymerase chain reaction (RT-qPCR). Moreover, their serum levels were also measured by a multiplex-bead-based immunoassay, and a histopathological study was performed. CYN produced immunomodulation mainly in the thymus of rats exposed to 75 μg CYN/kg b.w./day in both sexes. However, in the spleen only IL-1β and IL-2 (males), and TNF-α and IFN-γ (females) expression was modified after CYN exposure. Only female rats exposed to 18.75 μg CYN/kg b.w./day showed a significant decrease in TNF-α serum levels. There were no significant differences in the weight or histopathology in the organs studied. Further research is needed to obtain a deeper view of the molecular mechanisms involved in CYN immunotoxicity and its consequences on long-term exposures.

## 1. Introduction

Cylindrospermopsin (CYN) is currently the most studied cyanotoxin worldwide after microcystins (MCs) [1,2]. CYN has garnered increasing scientific interest because of its wide occurrence, bioaccumulation, and toxicity [3]. This alkaloid is mainly produced by *Cylindrospermopsis raciborskii* and *Chrysosporum ovalisporum* [4], and it is a stable tricyclic guanidine moiety connected by a hydroxylated bridging carbon to an uracil—the uracil moiety needed for its toxicity [5].

The occurrence of CYN and/or CYN-producing species has been reported worldwide, including temperate zones, and there is increasing concern that this toxin will represent serious human, as well as environmental, health risks across many countries [6]. Human exposure to CYN may occur by different pathways, the ingestion of drinking water being the most likely exposure route. Moreover, recreational activities in lakes with cyanobacterial blooms may also expose individuals intermittently to high concentrations of CYNs [5]. It has been demonstrated that cyanobacterial toxins, including CYN, are able to accumulate through the food chain [7]. To protect consumers from the adverse effects of CYN, provisional guideline values (GVs) in waters for CYN (lifetime drinking water GV, 0.7 µg/L; short term drinking water GV, 3 µg/L; and recreational exposure, 6 µg/L) have been recently proposed [5].

Inhibition of protein and glutathione synthesis are its best-known mechanisms of action [8,9], together with induction of oxidative stress [10,11] and genotoxicity [12]. Taking in to account the available studies, the liver, kidneys, and erythrocytes can be important target organs of CYN toxicity [5]. Even though other studies suggest that it is distributed to all major organs [13], including the lymphoid organs thymus and spleen, among those that may be affected [14,15,16].

Immunotoxicity can be defined as “any adverse effect on the immune system that can result from exposure to a range of environmental agents, including chemicals”, and it includes studies of different immune pathologies, such as immune dysregulation (suppression or enhancement), among others [17].

The immune system is specialized in defence against pathogens, and it is made up of cells and tissues dispersed throughout the body [18]. The organs which constitute the immune system and/or contribute to immune function consist of the bone marrow, the spleen, the thymus, and the lymph nodes, a network of lymphoid tissues along secretory surfaces and the skin [19]. The thymus and the bone marrow are the primary lymphoid organs, and the spleen and the lymph nodes, amongst others, constitute the secondary lymphoid organs [20]. The thymus is a significantly important organ to the immune system, which serves as the body’s defence mechanism, supplying surveillance and protection against diverse pathogens, tumours, antigens, and mediators of tissue damage [21]. The spleen is the biggest secondary lymphoid organ in the body and is the place of immune responses to blood-borne infections, which removes senescent cells and microbial particles from the blood [18,22].

The thymus produces hormones and cytokines that regulate immune function [23]. Cytokines have essential roles in the control of immune responses and their main function is to modulate inflammation, playing an important role in regulating the immune response, existing proinflammatory and anti-inflammatory cytokines [23,24]. Among cytokines, IL-1β, IL-6, TNF-α, and IFN-γ are considered as proinflammatory cytokines [25,26], while IL-2 is considered as anti-inflammatory [27]. IL-1β, together with IL-6 and TNF-α, act on distant target cells to initiate the acute phase response [18].

In recent years, the number of studies about the immunotoxic effects caused by cyanotoxins have grown significantly, mainly in the case of MCs. There is growing evidence that cyanotoxins may induce immunotoxicity, which should be considered due to the numerous negative changes that these disorders of immune functions may cause in organisms, including carcinogenesis [28]. However, although some authors have shown that CYN can possibly alter cells of the immune system and their functions, these studies are very scarce, as it has been recently reviewed by different authors [29,30]. Some studies have demonstrated necrosis and atrophy of the thymus and the spleen in rodents [14,15,31], followed by the first reports about the alteration of the human innate immune response in vitro due to the action of CYN [32,33,34]. Nonetheless, further in vivo studies with mammals with realistic exposure scenarios are required for a better understanding of the immunomodulation induced by CYN. Besides, the study of cytokine production and transcriptional factors would be useful to elucidate the molecular mechanisms underlying CYN immunotoxicity. In this sense, gene expression of cytokines by quantitative real-time polymerase chain reaction (RT-qPCR) is a frequently investigated parameter in most animal models, showing a dualistic response (immune-stimulative or immunosuppressive) after MCs exposure [30], but its investigation is still very scarce in the case of CYN, and limited to fish and murine cells [35,36].

Taking this into account, the aim of this work was to study for the first time the toxicity of pure CYN in the thymus and the spleen of male and female Sprague Dawley rats, orally exposed to different doses of CYN daily for 28 days, according to the Organisation for Economic Co-operation and Development (OECD) 407 guideline [37]. Alterations in gene expression of cytokines, such as interleukins (IL: IL-1β, IL-2, IL-6), tumour necrosis factor-alpha (TNF-α), and interferon-gamma (IFN-γ) in the thymus and the spleen, and their serum levels were investigated by RT-qPCR and multiplex assay, respectively. Moreover, organ weights and histopathological changes were recorded with the objective to complete the immunotoxicology profile of CYN.

## 2. Results and Discussion

Although the effects of CYN on the immune system have been scarcely studied, it is known that it can cause immunotoxic effects in in vitro and in vivo models [2,29,30]. In this study we show for the first time the immunotoxic effects that pure CYN can cause in male and female rats orally exposed by repeated doses for 28 days to 18.75, 37.5, or 75 μg CYN/kg b.w./day, following the OECD 407 guideline [37].

### 2.1. Gene Expression Analysis of ILs by RT-qPCR

**Figure 1:**
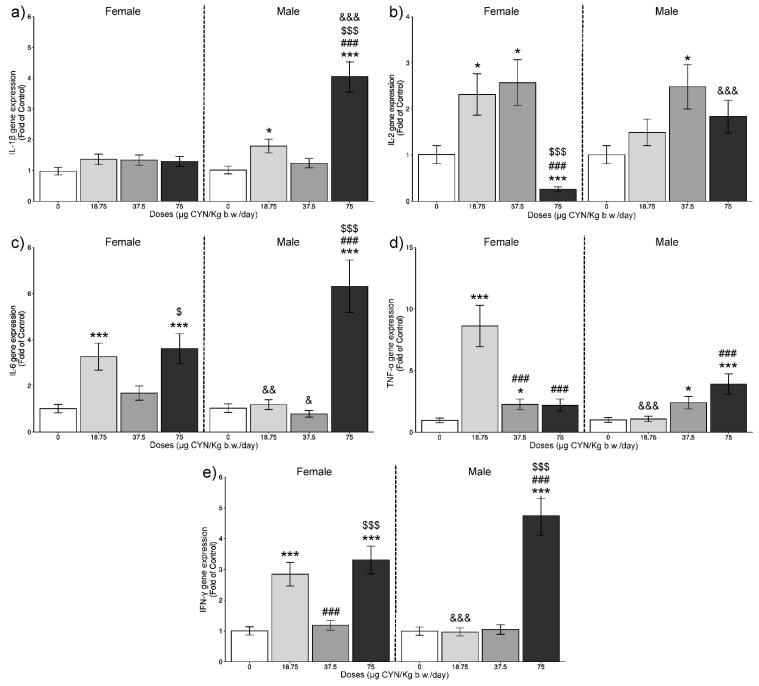
Interleukins (IL) IL-1β (**a**), IL-2 (**b**), IL-6 (**c**), tumour necrosis factor alpha (TNF-α) (**d**), and interferon-gamma (IFN-γ) (**e**) gene expression expressed as fold of control in the thymus of female and male rats in the different exposure scenarios (0, 18.75, 37.5, and 75 µg CYN/kg b.w./day, for 28 days). Mean values (±SEM) of N = 6 are calculated (* *p* < 0.05, ** *p* < 0.01, *** *p* < 0.001). The coded symbols represent the following: *—statistical differences compared with respective control group; ^#^—statistical differences compared with the 18.75 µg CYN/kg b.w./day dose group; ^$^—statistical differences compared with the 37.5 µg CYN/kg b.w./day dose group; ^&^—statistical differences between female and male rats exposed to the same dose.

**Figure 2:**
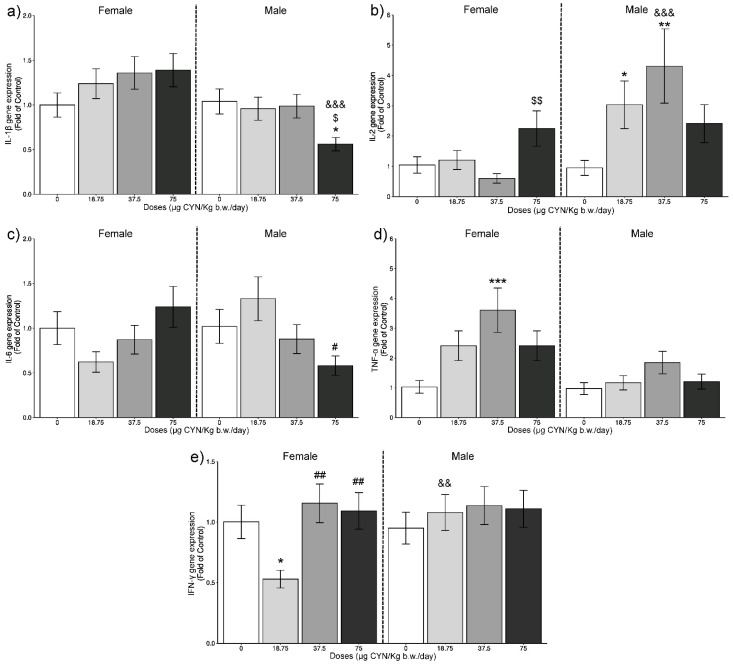
IL-1β (**a**), IL-2 (**b**), IL-6 (**c**), TNF-α (**d**), and IFN-γ (**e**) gene expression, expressed as fold of control in the spleen of female and male rats in the different exposure scenarios (0, 18.75, 37.5, and 75 µg CYN/kg b.w./day, for 28 days). Mean values (±SEM) of N = 6 are calculated (* *p* < 0.05, ** *p* < 0.01, *** *p* < 0.001). The coded symbols represent the following: *—statistical differences compared with respective control group; ^#^—statistical differences compared with the 18.75 µg CYN/kg b.w./day dose group; ^$^—statistical differences compared with the 37.5 µg CYN/kg b.w./day dose group; ^&^—statistical differences between female and male rats exposed to the same dose.

Results from the relative gene expression of the studied genes in the thymus and the spleen of rats exposed to CYN are represented in Figure 1 and Figure 2, respectively.

No alterations were observed in the gene expression of IL-1β (Figure 1a) in the thymus of females after repeated exposure to CYN at any dose assayed. In males, significant upregulations were shown at 18.75 and 75 μg CYN/kg b.w./day compared with its respective control group (*p* < 0.05 and *p* < 0.001, respectively). Moreover, significant overexpression was also observed at the highest dose compared with the lowest ones and to the female rats exposed to the same dose (*p* < 0.001).

These results are in agreement with those obtained by Sieroslawska et al. [35], which reported a significant increase in IL-1β gene expression of carp phagocytes after 24 h of exposure to CYN.

In relation to IL-1β expression in the spleen (Figure 2a), only the males showed significant differences. In this case, a decrease in the gene expression was observed at the highest dose compared with its respective control group, and to the medium dose (*p* < 0.05). Additionally, significant statistical differences were observed between males and females exposed to 75 µg/kg b.w./day (*p* < 0.001).

IL-1β is a molecule involved in the proinflammatory response, which is widely released in bacterial infections [38], and it is expressed in an extensive range of tissues and cells, specifically in macrophages in lymphoid organs, including the thymus and the spleen, among others [39].

Furthermore, it seems that IL-1β implicates upregulation of the pleotropic cytokine IL-6 [40]. This is in agreement with the results obtained in the present work, where both IL-1β and IL-6 (Figure 1a,c) showed a significant overexpression in the thymus of males at the highest dose assayed. Additionally, in the spleen of male rats, a similar response between these two ILs, in this case a downregulation, was observed (Figure 2a,c).

Concerning IL-2 in the thymus (Figure 1b), significant differences were found at all doses of exposure for females. At the lowest and medium doses, there was an increase in the expression (*p* < 0.05), whereas a downregulation was observed at 75 μg CYN/kg b.w./day (*p* < 0.001). Besides, at the highest dose, this reduction was also significant compared with the lowest doses (*p* < 0.001).

In the spleen, IL-2 expression was not altered in comparison to control in females (Figure 2b), and only a significant increase was observed at 75 µg/ kg b.w./day compared with the 37.5 µg CYN/kg b.w./day dose group (*p* < 0.01). In males, a significant overexpression was observed at the low and medium doses compared with its respective control group (*p* < 0.05 and *p* < 0.01, respectively). Moreover, statistical differences were reported between females and males exposed to 37.5 µg CYN/kg b.w./day (*p* < 0.001).

This cytokine plays an immunoregulatory role; it can incite the growth and development of immune cells in the initiation of the immune response, and keeps them alive as effector cells, but subsequently it has a pro-apoptotic effect [41]. Moreover, it modulates the expression of receptors for other cytokines and transcription factors, therefore either promoting or inhibiting cytokine cascades that can be related with each T helper differentiation state [42].

In the thymus, significant increases in IL-6 gene expression (Figure 1c) were shown at 18.75 and 75 μg CYN/kg b.w./day in female rats and only at 75 μg CYN/kg b.w./day in male rats compared with their respective control groups (*p* < 0.001). In females, this increase at the highest dose was statistically different compared with the 37.5 µg CYN/kg b.w./day dose group (*p* < 0.05). In males, the overexpression at the highest dose was also significant in comparison to the lowest doses (*p* < 0.001), and significant differences were observed compared with females exposed to 18.75 and 37.5 µg CYN/kg b.w./day (*p* < 0.01 and *p* < 0.05, respectively). IL-6 is a proinflammatory pleiotropic cytokine with effects on inflammation, haematopoiesis, and immune response, with important effects in adaptive immunity [41,43]. It is involucrated in mediating the activation of T and B cells, which are key cells in the pathogenesis of many autoimmune diseases [44].

Concerning IL-6 gene expression in the spleen (Figure 2c), no significant changes respect to the control were observed neither in females nor in males. A significant decrease was only observed in males exposed to the highest dose compared with the 18.75 µg CYN/kg b.w./day dose group (*p* < 0.05). In a similar way, no effects were observed in this proinflammatory cytokine after murine macrophage-like RAW 264.7 cells exposure to pure CYN (1 µM) for 24 h [36]; although, the experimental models and exposure conditions employed in both works are very different for a direct comparison.

In the case of TNF-α in the thymus (Figure 1d), significant upregulations were shown with 18.75 and 37.5 µg CYN/kg b.w./day (*p* < 0.001 and *p* < 0.05, respectively) in females and with 37.5 and 75 µg CYN/kg b.w./day (*p* < 0.05 and *p* < 0.001, respectively) in males, compared with their respective control groups. Moreover, in females the increase shown at 18.75 µg CYN/kg b.w./day was also significant compared with the other doses (*p* < 0.001) and to male rats (*p* < 0.001). In males, this alteration shown at the highest dose was significant compared with the lowest one (*p* < 0.001). In the spleen of female rats exposed to 37.5 µg CYN/kg b.w./day, there was also a significant increase in TNF-α gene expression, compared with its respective control group (Figure 2d), and it was even more pronounced (*p* < 0.001) than in the thymus, while significant differences in males at any of the doses assessed were not observed. Similar to our results, upregulation of TNF-α was reported after exposure of phagocytic cells from *Cyprinus carpio* to CYN [35], and in RAW 264.7 macrophages exposed to CYN [36].

It has been described that the metabolic effects of TNF-α include a decrease in cholesterol and an increase in triglycerides, as well as hyperglycaemia [45]. In this sense, the increases in TNF-α expression observed in this work are in agreement with the significant decreases in cholesterol levels reported previously in our lab in female Sprague Dawley rats after exposure to 18.75, 37.5, and 75 µg CYN/kg b.w./day for 28 days [46]. Moreover, we also reported an increase in triglycerides and glucose levels after exposure to 18.75 and 37.5 µg CYN/kg b.w./day. TNF-α is one of the most important cytokines in the immune system with pleiotropic effects on normal and malignant cells [45,47].

This inflammatory cytokine has shown to have a significant role in the regulation of thymocyte production, among other functions [23]. Although at first it was considered as a proinflammatory molecule, it has been reported that it also mediates a paradoxical anti-inflammatory and immunomodulatory effect [48]. TNF-α also stimulates the production of IL-6 [23]. This agrees with the correlation observed in the present work between the gene expression of these two molecules in the thymus of female and male rats exposed to 18.75 and 75 µg CYN/kg b.w./day, respectively.

In relation to IFN-γ in the thymus (Figure 1e), significant increases in its gene expression were observed at 18.75 and 75 µg CYN/kg b.w./day (*p* < 0.001) in females and at 75 µg CYN/kg b.w./day (*p* < 0.001) in males, compared with their respective control groups. In the case of females, significant differences were observed among the 3 doses assayed (*p*< 0.001), and at the lowest dose compared with the males (*p* < 0.001). For males, the increase shown at 75 µg CYN/kg b.w./day was also significant versus the lowest and medium doses. Previous studies reported that IL-2 has an important role in the synthesis of IFN-γ [49], in agreement with the upregulation of both genes found in the present work in the thymus of female rats exposed to 18.75 μg CYN/kg b.w./day.

In the spleen, a significant decrease in IFN-γ gene expression (Figure 2e) occurred in females exposed to 18.75 µg CYN/kg b.w./day compared with the control group (*p* < 0.05), this also being significant compared with males (*p* < 0.01). Besides, a significant upregulation was observed at 37.5 and 75 µg CYN/kg b.w./day compared with 18.75 µg CYN/kg b.w./day dose (*p* < 0.01).

IFN-γ is often considered as a main effector of immunity, playing a key role in the activation of cellular immunity and consequently stimulation of antitumor immune response [26,50]. This cytokine is an important coordinator of innate and adaptive immunity, which in an inflammatory process unleashes the activation of the immune response and stimulates the elimination of pathogens, and it also prevents overactivation of the immune system and tissue damage [50,51]. In our study, there is a direct relation between the gene expression of IL-6, IFN-γ, and TNF-α in the thymus of female rats exposed to 18.75 µg CYN/kg b.w./day and in male rats exposed to 75 µg CYN/kg b.w./day, showing an upregulation. Besides, there was also an overexpression of IL-6 and IFN-γ in the thymus of female rats exposed to the highest dose. In this sense, previous works reported a stimulation of the secretion of IL-6 by thymic epithelial cells mediated by IFN-γ [23]. IFN-γ are among the critical cytokines that initiate the downstream signalling cascade to secrete TNF-α and IL-2, among others [52]. In this sense, we observed the same behaviour in the thymus of female rats exposed to 18.75 µg CYN/kg b.w./day for IL-2, TNF-α, and IFN-γ gene expression.

As general observation, the thymus seems to suffer from a more extensive alteration of ILs gene expression in comparison to the spleen. This could be due as thymus is the first lymphoid organ that shows morphologic alterations after exposure to many immunotoxic agents [53]. Additionally, it has been reported for other toxicants that thymus can show a higher ability to the xenobiotic uptake [54]. In regard to the sex, a clear sex-related pattern is not evident. Thus, for example, at the highest dose, in the thymus, the gene expression levels of IL-1β were higher in males while in spleen the contrary response was observed. Additionally, at the lowest dose IFN-γ showed higher expression values in females in the thymus, whereas, in the spleen, the males show the greatest ones.

Although changes of ILs gene expression induced by CYN have been scarcely investigated, this is not the case for other cyanotoxins. For microcystins (microcystin-LR, MC-LR), the studies on mammals are limited. Thus, Cao et al. [55] evaluated the expression level of inflammatory-related factors in jejunum. Mice were treated with different doses (1, 30, 60, 90, and 120 µg/L) of MC-LR for 6 months. The microstructure and mRNA expression levels of inflammation-related factors in jejunum were analysed. Results showed that the microstructure of the jejunum was destroyed and expression levels of inflammation-related factors IL-1β, IL-8, TNF-α, and IL-10 were altered at different MC-LR concentrations. In fish, however, the number of available studies is higher [30]. The general trend for these studies is an immune-stimulative response at low MC concentrations and an immunosuppressive answer at high MC levels.

### 2.2. Cytokine Serum Levels

Alterations in cytokine levels (pg/mL) were measured in serum of male and female rats exposed to different doses of CYN (Table 1).

In general, compared with the control group, there are only significant differences in females in the case of TNF-α at the lowest dose, showing a significant decrease in the levels of this cytokine (** *p* < 0.01). However, a significant increase was found in levels of IL-2, IL-6, IFN-γ (^&&^ *p* < 0.01), and TNF-α (^&^ *p* < 0.05) in males compared with females exposed to the same dose (37.5 µg CYN/kg b.w./day).

In females, the general trend for the different cytokines assessed is a decrease in their levels at the doses of 18.75 and 37.5 µg CYN/kg b.w./day with respect to the control group, to increase again at the highest dose (75 µg CYN/kg b.w./day) compared with the lowest doses, although there were not statistically significant in comparison to the control group. Furthermore, there are significant differences between sexes for all the cytokines assessed at the medium dose (37.5 µg CYN/kg b.w./day) except for IL-1β, all these levels being higher in males than in females.

IL-1β negatively regulates the responses mediated by IFN-γ, by induction of Cyclooxygenase-2 and prostaglandin E2 expression, leading to the suppression of IFN-γ production [38]. This could explain the trend in the present work in females exposed to 37.5 µg CYN/kg b.w./day with respect to the control group, where IL-1β serum levels show a slight increase, whereas IFN-γ is reduced in this group compared with the control levels.

The increasing trend in TNF-α serum levels in males in all doses, although not being statistically significant, may be a result of the significant increases in this cytokine expression in the thymus of the same individuals. A sporadic case could be the significant decrease in TNF-α serum levels in females exposed to 18.75 µg CYN/kg b.w./day compared with the control group, because TNF-α showed a significant overexpression in the thymus, and a tendency to increase in the spleen of these female rats. These contradictory effects are not related with the dose and would require further research.

Regarding the effects of other cyanotoxins, different authors [56,57,58] evaluated the ILs serum levels induced by microcystin-LR (MC-LR) using mice or rabbits as experimental model. Thus, in mice exposed to MC-LR ranging from 3.125 to 25.000 ug/kg b.w./d i.p. for 7 days, the level of TNF-α decreased with the increase in toxin dosage, while the levels of IL-6 increased with the MC-LR exposure doses showing a statistical difference from the control group, and no changes were detected on IL-10 levels [57]. In mice exposed intraperitoneally (i.p.) to 10 ug/kg.b.w. MC-LR day for 15 days, the toxin significantly enhanced the levels of osteoclastogenic cytokines (IL-6, IL-17, and TNF-α) in serum along with a simultaneous decrease in the levels of anti-osteoclastogenic cytokines (IL-10 and IFN-γ) [58]. MC-LR was able to disturb the rabbit immune system and a time–dose response relationship was found: the cytokine production in the low dose group (12.5 µg MC-LReq/kg) increased (TNF-α, IFN-γ, IL-4, IL-3, and IL-6), but began to decrease after 24 h compared with the control. At the high dose group (50 µg MC-LReq/kg), the production of all the evaluated ILs decreased [56].

### 2.3. Organ Weights and Histopathology

Results obtained for absolute organ weights are presented in Table 2. No significant changes were observed in the thymus or the spleen of both sexes at any concentration assessed. In relation to organ weight/body weight ratio of rats exposed to CYN (Table 2), the only significant difference (*p* < 0.05) was found in the spleen of male rats exposed to the highest dose (75 μg CYN/kg b.w./day). However, no alterations were observed in the thymus of these rats. In relation to the relative organ weight/brain weight, no significant changes were observed in male or female rats exposed to CYN. Similarly, Humpage and Falconer [59] exposed mice up to 240 µg/kg/d CYN-containing cyanobacterial extract in their drinking water for 11 weeks, and they did not find changes in the weight of spleen and thymus, but in other organs, such as liver and kidney.

**Figure 3:**
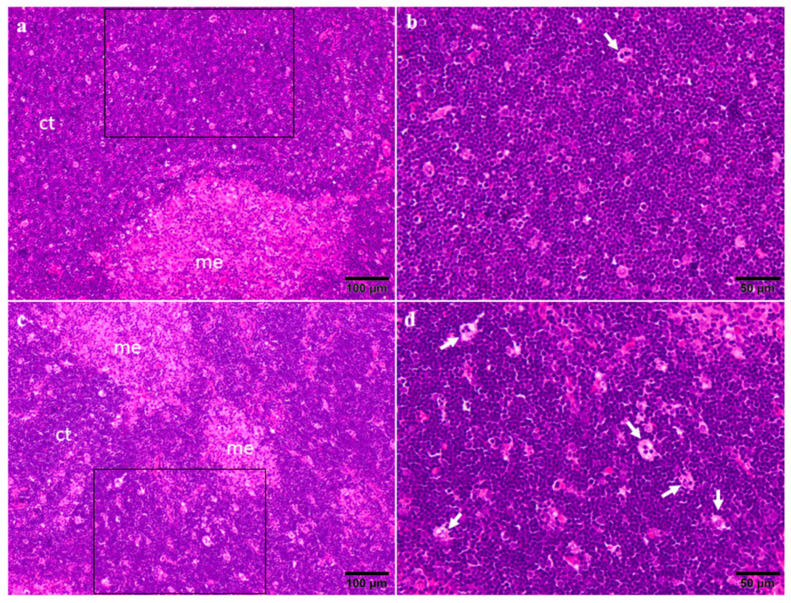
Representative photomicrographs of the thymus from control (**a**,**b**) and 75 μg CYN/kg b.w./day groups (**c**,**d**). b and d images are higher magnifications of the fields framed in black (a and b, respectively). Notice the frequent presence of tingible body macrophages (arrow) in the CYN group. me—medulla; ct—cortex.

The thymus from both the control and 75 μg CYN/kg b.w./day groups showed similar relation between cortex and medulla (Figure 3a,c). In the cortex of the thymus in control rats there were infrequently large macrophages containing phagocytosed apoptotic bodies (termed as tingible body macrophages), indicative of apoptosis (Figure 3b); meanwhile, in the CYN-administered rats, these apoptotic bodies were slightly more frequent (Figure 3d). These results are in agreement with the obtained by Terao et al. [14], which reported necrosis of lymphocytes in the cortical layer of the thymus of mice exposed to CYN isolated from *Umezakia natans* by intraperitoneal (IP) injection (0.2 mg/kg b.w.). Later, Seawright et al. [15] observed atrophy of the thymic cortex in mice orally exposed to a *C. raciborskii* culture containing 0.2% CYN. Furthermore, thymic atrophy was observed in mice after a single oral dose of *C. raciborskii* cellular suspension [31]. All these studies were performed with cyanobacterial extracts, and these may contain more bioactive compounds other than CYN that increase the toxicity, as it has been previously reported in various works [6,11,60,61,62,63,64]. On the contrary, Chernoff et al. [65] exposed mice orally for 90 days to 75–300 µg/kg/d purified CYN and they did not observe histopathological lesions in thymus.

**Figure 4:**
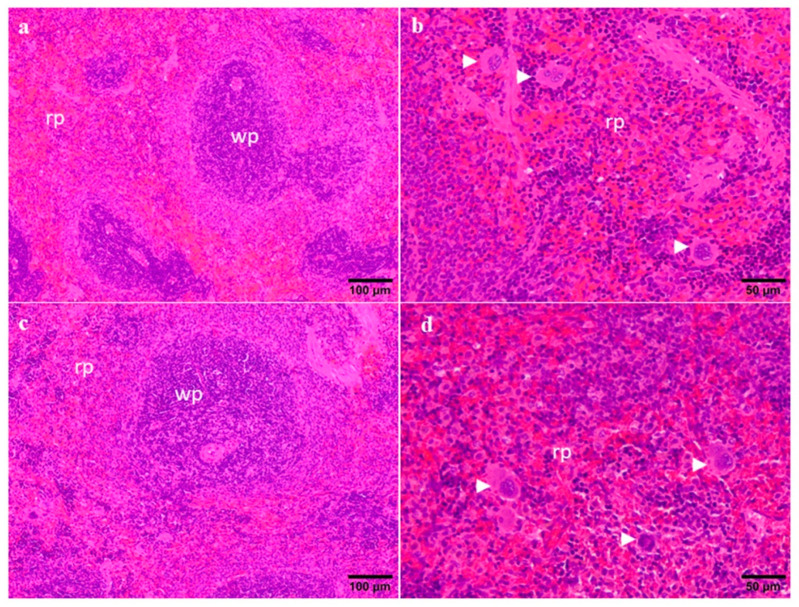
Representative photomicrographs of the spleen from control (**a**,**b**) and 75 μg CYN/kg b.w./day groups (**c**,**d**). b and d images are higher magnifications of the red pulp from the images a and b, respectively. Notice the normal presence of megakaryocytes (arrowhead) in both the control and CYN group. wp—white pulp; rp—red pulp.

The observation of the spleen did not reveal any change associated with the highest dose of CYN compared to the control rats (Figure 4). No difference between either males or females was found. The samples from both groups showed the normal architecture of the spleen displaying a well-developed white pulp (Figure 4a,c), and a red pulp with mild extramedullary haematopoiesis characterized by the presence of megakaryocytes that are commonly seen in the red pulp of healthy rodents in a low number (Figure 4b,d). On the contrary, histopathological alterations were reported in the white pulp of the spleen of mice orally exposed to *C. raciborskii* culture [15]. Moreover, in mice exposed to purified CYN (IP) or cellular suspension of *C. raciborskii* by gavage, a characteristic of these animals was the appearance of lymphophagocytosis in the spleen, which is indicative of immunotoxic response [31]. Other authors, however, did not observe histopathological lesions in spleen in mice orally exposed for 90 days to 75–300 µg/kg/d purified CYN [65]. Therefore, these studies suggested that the thymus and the spleen are not primary targets of CYN exposure, in agreement with our results.

These contradictory results could be due to several factors: the exposure route, such as the IP or gavage routes [14,15,31]—whereas, in our study, oral exposure through the diet by gelatine tablets was used; the experimental model (mice/rats); the time of exposure (single IP dose [14]/single oral dose by gavage [15,31]/repetitive IP daily doses for 14 days [19]/repetitive oral doses by ingestion for 28 days). Moreover, in our study the effects are due to the pure toxin (CYN), in contrast with those obtained with cyanobacterial extracts or cellular suspensions of cultures containing not only CYN but also other active substances [14,15,31].

In general, the results obtained in this work evidence the sensitivity of molecular biomarkers in the study of CYN’s immunotoxicity. Thus, changes observed in the gene expression of different ILs do not result in altered IL serum levels or clear histopathological damage of lymphoid organs. These results agree with the findings of Diez-Quijada et al. [46], who performed a repeated-dose, 28-day oral study in rats and did not observe changes in most of the parameters investigated; although, they did observe a very mild affectation of the liver and the kidney at the highest dose. The authors concluded that longer oral toxicity studies were necessary to define the potential consequences of long-term CYN exposure. Thus, as it is well known, liver and kidney are the primary targets of CYN exposure, and this is not the case for the immune system; although, it does suffer from alterations of ILs gene expression, that could be indicative of an immunotoxic response.

## 3. Conclusions

The present work shows for the first time the immunotoxic effects caused by pure CYN in the thymus and the spleen of male and female Sprague Dawley rats orally exposed to 18.75, 37.5, and 75 μg CYN/kg b.w./day for 28 days. Alterations in gene expression were found principally in the thymus at the highest dose evaluated (75 μg CYN/kg b.w./day) in males (IL-1β, IL-6, TNF-α and IFN-γ) and in females (IL-2, IL-6, IFN-γ). Moreover, there were no statistically significant differences in serum levels of cytokines between treated rats and control groups, except for females in the case of TNF-α at the lowest dose. No significant differences were found in the weights of the thymus and the spleen of the exposed rats, and no histopathological changes were found when compared to their respective control groups. These results highlight the need to fulfil further research to determinate the immunotoxicity caused by long-term exposure to CYN and elucidate the mechanisms involved.

## 4. Materials and Methods

### 4.1. Chemicals and Reagents

CYN standard with 95% purity was purchased from Enzo Life Sciences (Lausen, Switzerland). Neutral gelatine (powder) from pork protein was used as vehicle for toxin administration, and was supplied by Jesús Navarro S.A. (Alicante, Spain). Reagents for RT-qPCR and Bio-Plex Pro™ Rat Cytokine assay kit for cytokine serum levels were obtained from Bio-Rad Laboratories (Hercules, CA, USA) and Qiagen (Madrid, Spain). The rest of the chemicals were purchased from Sigma-Aldrich (Madrid, Spain).

### 4.2. Animal Housing and Experimental Set-Up

The subchronic toxicity study (28 days) was performed at the Central Service of Experimental Animals (SAE) of the University of Córdoba (Spain), under the code 20-CAM-01 in conformity with the OECD Guideline 407 [37]. All animals received human care in agreement with the Directive for the protection of animals used for scientific purposes [66,67,68], and all methods have been authorized by the Ethical Animal Experimentation Committee of the University of Córdoba and by the Junta de Andalucía (project no. 20-03-2017/047).

For the experimental set up, Sprague Dawley rat strain RjHan:SD (24 male and 24 female) were supplied by Janvier Labs (Le Genest-Saint-Isle, France) and were treated according to Diez-Quijada et al. [46]. Briefly, after a 1-week acclimation period (room temperature 22 ± 3 °C, 50–60% of relative humidity, 12 h light/dark cycle), when rats were fed with standard laboratory diet (Harlan Laboratories, Barcelona, Spain) and water ad libitum, they were randomly assigned to the control and dose groups (6 rats/sex/group). The sample size, six females and six males for each dose level, was chosen according to the OECD 407 guideline [37]. At this point, rats were 5 weeks old, and their average body weights (b.w.) were 173.5 ± 6.24 g (males) and 155.3 ± 3.10 g (females).

### 4.3. Experimental Exposure

For toxin administration, individual dietary dose formulations were prepared every day during the 28 days experiment. For this purpose, the volume (µL) of CYN standard to be incorporated in 3 mL of neutral gelatine was calculated to provide the selected doses and let to solidify overnight at 4 °C, as described in Diez-Quijada et al. [46]. This method of oral administration by gelatine tablets allows a better control of dosage, and eliminates pain and distress in animals compared with other methods, such as gavage [69].

The doses were selected according to a previous 90-day oral CYN exposure study performed in mice [65], where 75 µg CYN/kg b.w. induced several biochemical and histopathological alterations. So, this was fixed as the highest dose in the present study and, using a descending factor of two, according to the OECD Guideline 407 [37], the selected doses were 18.75, 37.5, and 75 µg CYN/kg b.w./day.

### 4.4. RNA Extraction and Reverse Transcription

For extraction and purification of total RNA, the RNeasy Mini Kit™ (catalog number: 74104, Qiagen, Madrid, Spain) was used following the manufacturer’s instructions. During RNA purification, on-column DNase digestion was also performed with the RNAse-free DNase set (catalog number 79254). RNA purity was assessed as the 260/280 nm absorbance ratio using a NanoDrop 2000 (Thermo Scientific, Pittsburgh, PA, USA). The RNA was then stored at −80°C until reverse transcription (RT). cDNA was synthesized from 2 μg of total RNA using QuantiTect^®^ reverse transcription kit (catalog number: 205311, Qiagen, Madrid, Spain) in 28 μL reaction volume, as described by the manufacturer. The cDNA was stored at −20 °C until further use.

### 4.5. Gene Expression Analysis by Quantitative Real-Time PCR (RT-qPCR)

A RT-qPCR protocol was developed to measure the mRNA levels for the five tested cytokines in the thymus and the spleen of rats, using the housekeeping gene Glyceraldehyde 3-phosphate dehydrogenase (*GAPDH*) as internal control. The cDNA obtained in the previous step was diluted (1:1.5) in RNase-free water and amplified by the PCR in a final reaction volume of 20 µL (96-well plate), which also includes the primePCR probe assay for the corresponding cytokine and the iTaq universal probes Supermix (catalog number 1725134). Amplification was performed in a LightCycler^®^480 System (Roche, Berlin, Germany) according to the following parameters: 95 °C for 2 min followed by 50 cycles of 95 °C for 5 s and 60 °C for 30 s. Amplification data were collected by the sequence detector and analyzed with sequence detection software supplied by the manufacturer. The RNA concentration in each sample was determined from the threshold cycle (Ct) values and calculated with the sequence detection software supplied by the manufacturer. For the treated samples, evaluation of 2^−ΔΔCT^ indicates the fold change in mRNA expression relative to the internal control *GAPDH*, and to the untreated control, used as the calibrator [70].

### 4.6. Multiplex Assay

At the end of the 28-day period, blood samples were obtained by intracardiac injection from the rats fasted overnight and anesthetized. Levels of five cytokines (IL-1β, IL-2, IL-6, TNF-α, and IFN-γ) were measured in serum samples using a Bio-Plex Pro™ Reagent Kit V with Flat Bottom Plate (catalog number: 12002798) and the different Bio-Plex Pro™ Rat Cytokines Sets, following the manufacturer’s instructions (Bio-Rad Laboratories, Inc., Hercules, California, USA). After dilution and mixture of the coupled magnetic beads for the 5 cytokines, they were added (50 μL) into each well of the 96-well, flat-bottom assay plate supplied, and washed twice with Bio-plex wash buffer (100 μL). Then, the blank, and 4-fold serial dilutions of the 8 standard and the samples (50 μL) were added to the corresponding wells, followed by incubation with constant shaking (850 ± 50 rpm, 1 h, room temperature), and washing 3 times (100 μL). Diluted (1×) biotinylated detection antibodies (25 μL) (IL-1β, IL-2, IL-6, TNF-α, and IFN-γ) were also added to the wells, incubated for 30 min and washed 3 times (100 μL). Finally, streptavidin–phycoerythrin (SA-PE) (50 μL) was added to the wells, incubated for 10 min, and washed 3 times (100 μL). At the end, beads were resuspended in assay buffer (125 μL), incubated for 30 s, and the plate was read on a Bio-Plex^®^ 200 Multiplex system (Bio-Rad Laboratories Inc., Hercules, CA, USA) available at the Biology Service of the Centro de Investigación, Tecnología e Innovación (CITIUS) of the University of Sevilla. All washes were performed with Bio-Plex wash buffer using the Bio-Plex^®^ Handheld Magnetic Washer (catalog number 171020100), and incubation steps performed in constant shaking, as described above. For readings with the Bio-Plex 200 instrument, the following settings were set: RP1 (PMT) High; DD Gates: 5000 (low) and 25,000 (high); Bead Events: 50. Data from the multiplex analysis was obtained using the Bio-Plex Manager™ version 4.1.1 software (Bio-Rad Laboratories, Inc., Hercules, CA, USA).

### 4.7. Organ Weights and Histopathology

At the end of the study, and after the sacrifice of the animals, which was performed as described in Diez-Quijada et al. [46], the thymus and the spleen of all rats were macroscopically analysed, extracted, and weighed. At postmortem examination, tissue samples were immersed in 10% neutral buffered formalin for 24 h, routinely processed using an automatic processor, and embedded in paraffin wax. Sections of 4 μm were stained with haematoxylin and eosin to carry out the histological examination. Samples of the spleen and the thymus from the control and the highest dose group (75 μg CYN/kg b.w./day) were examined in a Modular Microscopy BX43 with a camera XC50 (Olympus, Shinjuku, Tokyo, Japan).

### 4.8. Statistical Analysis

Organ weights, and organ weights ratios were determined by sex and dose group, statistically analysed, and summarized by mean and standard deviation (SD). One-way analysis of variance (ANOVA) was performed to evaluate potential differences in the different variables studied. The Kolmogorov–Smirnov test was applied to check for normality assumption. Comparisons were made with the Tukey–Kramer multiple comparisons test (when statistically significant) or with the Kruskal–Wallis test, followed by Dunn’s multiple comparison tests (non-normality). Differences were considered significant from * *p* < 0.05. All the statistical analysis were conducted using GraphPad InStat software (GraphPad Prism 9 Software Inc., La Jolla, CA, USA).

IL-1β, IL-2, IL-6, TNF-α, and IFN-γ gene expression were analyzed by fitting general linear models (GLMs) with the explanatory variable’s doses of CYN (control, 18.75, 37.5, and 75 μg CYN/Kg b.w./day), sex (female vs. male), and the interaction between them. Gene expression, being a continuous strictly positive variable, was fitted to a gamma distribution with a log link function. After model adjustment, whenever a main factor was found significant, marginal post hoc Tukey tests were performed to check significant differences between the control and doses within the same sex, and between sexes subjected to the same dose of CYN. All GLMs and posthoc tests were run with the package base and emmeans [71] and plotted with ggplot2 [72] in R 4.1.2 [73].

## Figures and Tables

**Table 1:** Serum levels (pg/mL) of cytokines (IL-1β, IL-2, IL-6, TNF-α, and IFN-γ) of male and female rats fed with 18.75, 37.5, and 75 µg CYN /kg b.w./day for 28 days. Values are mean ± SD for 6 rats/sex/group. The differences between control and treated groups for male and female rats were evaluated by the Kruskal–Wallis test (K.W.). The significance level observed are ** *p* < 0.01 in comparison to control group values, and ^&^ *p* < 0.05, ^&&^ *p* < 0.01 comparing males and females exposed to the same dose.

MALE						FEMALE			
		Group 1 (0 µg/Kg/day)	Group 2 (18.75 µg/Kg/day)	Group 3 (37.5 µg/Kg/day)	Group 4 (75 µg/Kg/day)	Group 1 (0 µg/Kg/day)	Group 2 (18.75 µg/Kg/day)	Group 3 (37.5 µg/Kg/day)	Group 4 (75 µg/Kg/day)
		N = 6	N = 6	N = 6	N = 6	N = 6	N = 6	N = 6	N = 6
IL-1β	MEAN	192.01	205.92	236.28	169.93	218.09	190.12	262.53	300.43
	SD	45.21	56.19	56.98	33.89	41.22	61.96	159.78	146.09
	K.W χ^2^ = 7.825 *p* = 0.35; N.S.								
IL-2	MEAN	7587.473	9125.263	10128.74 ^&&^	9417.15	8988.63	4805.75	5649.99	8134.93
	SD	984.78	1312.51	735.55	1131.08	1147.15	1670.85	1533.65	1596.87
	K.W χ^2^ = 31.053 ^&&^ *p* < 0.01								
IL-6	MEAN	1027.99	1123.73	1369.89 ^&&^	1202.74	1172.01	637.66	782.97	1092.62
	SD	187.34	93.79	137.42	126.98	179.73	191.24	135.18	222.13
	K.W χ^2^ = 31.492 ^&&^ *p* < 0.01								
TNF-α	MEAN	653.40	786.23	908.90 ^&^	837.63	845.67	358.23 **	487.09	784.34
	SD	66.40	141.69	112.18	151.93	91.41	124.78	174.91	107.19
	K.W χ^2^ = 31.287 ** *p* < 0.01; ^&^ *p* < 0.05								
IFN-γ	MEAN	509.18	520.04	617.72&&	546.59	551.73	330.11	364.92	480.6
	SD	106.23	40.34	64.04	45.84	79.45	92.84	68.47	96.20
	K.W χ^2^ = 29.699 ^&&^ *p* < 0.01								

K.W—Kruskal–Wallis test; N.S.— not significant. The significance level observed are ** *p* < 0.01 in comparison to control group values, and ^&^ *p* < 0.05, ^&&^ *p* < 0.01 comparing males and females exposed to the same dose.

**Table 2:** Absolute organ weight, relative organ weight/body weight, and relative organ weight/brain weight of the thymus and the spleen of male and female rats fed with 18.75, 37.5, and 75 µg CYN /kg b.w./day for 28 days. Values are mean ± SD for 6 rats/sex/group. The differences between control and treated groups for male and female rats were evaluated by ANOVA test (F values) or by the Kruskal–Wallis test (K.W.). The significance level observed is * *p* < 0.05 in comparison to control group values.

**ORGAN WEIGHT DATA SUMMARY**											
**MALE**						**FEMALE**					
		Group 1	Group 2	Group 3	Group 4			Group 1	Group 2	Group 3	Group 4
		(0 µg/Kg/day)	(18.75 µg/Kg/day)	(37.5 µg/Kg/day)	(75 µg/Kg/day)			(0 µg/Kg/day)	(18.75 µg/Kg/day)	(37.5 µg/Kg/day)	(75 µg/Kg/day)
		N = 6	N = 6	N = 6	N = 6			N = 6	N = 6	N = 6	N = 6
THYMUS (g)	MEAN	1.1	1.0	1.0	1.1	THYMUS (g)	MEAN	0.7	0.8	0.9	0.9
	SD	0.1	0.1	0.2	0.1		SD	0.0	0.2	0.1	0.1
F (20.3) = 1.276 *p* = 0.31; N.S.						F (20.3) = 2.745 *p* = 0.07; N.S.					
SPLEEN (g)	MEAN	1.1	1.1	1.1	1.2	SPLEEN (g)	MEAN	0.6	0.7	0.7	0.7
	SD	0.1	0.1	0.2	0.1		SD	0.0	0.1	0.1	0.0
F (20.3) = 2.118 *p* = 0.13; N.S.						F (20.3) = 1.808 *p* = 0.18; N.S.					
**ORGAN WEIGHT/BODY WEIGHT RATIO DATA SUMMARY**											
**MALE**						**FEMALE**					
THYMUS (g)	MEAN	0.27	0.25	0.25	0.28	THYMUS (g)	MEAN	0.30	0.29	0.34	0.36
	SD	0.02	0.02	0.04	0.02		SD	0.01	0.06	0.04	0.04
F (20.3) = 1.913 *p* = 0.16; N.S.						F (20.3) = 3.681 *p* = 0.06; N.S.					
SPLEEN (g)	MEAN	0.25	0.26	0.26	0.31 *	SPLEEN (g)	MEAN	0.26	0.25	0.27	0.29
	SD	0.02	0.02	0.05	0.02		SD	0.02	0.04	0.02	0.02
F (20.3) = 3.795 * *p* < 0.05						K.W χ^2^ = 5.410 *p* = 0.14; N.S.					
**ORGAN WEIGHT/BRAIN WEIGHT RATIO DATA SUMMARY**											
**MALE**						**FEMALE**					
THYMUS (g)	MEAN	54.7	51.8	48.0	54.4	THYMUS (g)	MEAN	37.7	40.3	46.1	45.6
	SD	3.1	4.8	9.2	4.3		SD	3.5	7.9	7.5	6.3
F (20.3) = 1.702 *p* = 0.20; N.S.						F (20.3) = 2.366 *p* = 0.10; N.S.					
SPLEEN (g)	MEAN	50.9	55.1	51.3	60.6	SPLEEN (g)	MEAN	33.6	35.1	36.4	36.7
	SD	3.9	3.4	9.6	6.8		SD	3.7	4.6	1.7	1.5
F (20.3) = 2.916 *p* = 0.06; N.S.						F (20.3) = 1.216 *p* = 0.33; N.S.					

F—statistics ANOVA test; K.W.—Kruskal–Wallis test; N.S.—not significant. The significance level observed is * *p* < 0.05 in comparison to control group values.

## Data Availability

Not applicable.
